# The influence of gender and sport on popliteal angle and dorsiflexion in junior high school students

**DOI:** 10.1186/s12891-024-07429-7

**Published:** 2024-05-19

**Authors:** Krzysztof Pietrzak, Artur Bania, Krzysztof Nowocień, Bartosz Kraszewski, Marzena Wiernicka

**Affiliations:** 1 Pediatric and Adult Orthopedics Poland, Dolna Wilda 88G/104, 61-501 Poznań, Poland; 2 PhysioCenter, Osiedle Zwycięstwa 124, Poznań, Poland; 3 The Orthopedic Department, Poznań Multidisciplinary Municipal Hospital, Poznań, Poland; 4 Public Health Faculty, Poznań University of Medical Sciences, Poznań, Poland; 5 Department of Kinesiotherapy and Developmental Physiotherapy, Poznan University of Physical Education, Poznań, Poland

**Keywords:** Junior high school students, Gender, Range of motion

## Abstract

**Background:**

The aim of the study was to assess factors affecting the popliteal angle and foot dorsiflexion, in particular gender. The subjects were 142 students from the 2nd and 3rd year of Poznań junior high schools.

**Methods:**

The participants included 57 girls and 87 boys. Three raters examined each subject: a specialist in orthopaedics, a resident doctor and a physical therapy student.

Foot dorsal flexion was tested in a supine position with lower limbs extended. Next, dorsal flexion was evaluated with the knee and hip in 90 degrees of flexion. Finally, a passive knee extension (PKE) test was carried out. The significance of the PKE test is that the lower the angle the more flexible the hamstrings. This is because the PKE measurement is the distance to the right angle, that is a full knee extension with the hip flexed.

**Results:**

The non-parametric test (Mann–Whitney) and the Student’s t-test showed differences between the female and male gender in the measurements of the popliteal angle (*p* < .05000). The correlation was negative, which means that the hamstrings are more flexible in girls. No differences were found between gender and passive foot dorsiflexion and dorsiflexion with a flexed hip and knee. No differences were found between the group with the extended PE curriculum and the group with the standard number of PE classes in the range of motion of foot dorsiflexion and the value of the popliteal angle.

**Conclusions:**

Girls between 13 and 15 years old have a significantly larger hamstring flexibility, which is confirmed by the tests of the popliteal angle. No differences were found in dorsiflexion between girls and boys who have not been trained using a training model.

## Introduction

The human body undergoes changes throughout lifetime. The biggest changes in the shape of the feet and lower limbs as well as the range of motion in the joints take place in childhood and adolescence. This change is affected by a number of factors, such as hypermobility, increasing strength of the joint-stabilizing muscles as well as a possible relative shortening of the muscles with respect to the length of the bones in the lower limbs. The best approach in the different stages of development has been a subject of some controversy, not only in literature, but also in medical, physical therapy and coaching practice. One of these important stages is adolescence. This is the time when gender and exercise have a significant impact on developmental processes.

There has been considerable debate in the literature about the causes of a higher incidence of knee injuries in women, or whether the difference exists at all [1, 2]. There is also controversy as to whether women in certain age groups have more flexible hamstrings [3, 4]. We assumed that a higher hamstring flexibility in women may lead to knee hyperextension, which in turn decreases the stability of the joint and increases the risk of injury. However, a knee joint may be prone to injury not only due to imbalances in the flexibility and strength of the anterior and posterior thigh muscles [5, 6]. Imbalances between the individual parts of the hamstring muscles, which are more frequent in women, may also be a contributing factor [5, 6].According to a number of authors, in some sports women have a higher risk of ACL injury (for soccer and basketball there is a three-fold increase in the risk). Injury prevention programmes for women seem to be effective in soccer, but not basketball [7–9]. A higher number of injuries may also be caused by a general lack of awareness that activity is needed to prevent such injuries, and that women generally have a higher risk of ACL injury [10–14].

The objective of the study was to evaluate the effects of gender and sport on the popliteal angle and foot dorsiflexion in a representative group of junior high school students from Poznań. This is a continuation of a study on a patient cohort which we previously analyzed for other parameters. Both the grouping in the cohort and the research methods are different than in the previous studies [15, 16]. As traditional gender roles are changing, more and more women want to be professional athletes. A range of studies indicate that female athletes are more prone to injuries than male athletes [1, 2], which may be caused by the flexibility of the hamstrings. We also analysed if the number of PE classes in the curriculum affects these parameters, which also included performing calculations separately for each gender.

## Material and methods

The participants were 142 (57 female, 85 male) junior high school students Poznań (Poland), (age 13–15, mean ± SD 13.8 ± 1.0).

Sixty subjects were from classes with an extended physical education (PE) curriculum while 82 were from classes following the standard curriculum. The latter students described their level of physical activity as “non-active” or limited to PE classes at school. The students from the standard curriculum classes had 4 PE classes per week while those with the extended PE curriculum had between 14 and 18 PE classes per week. The sports included football, basketball and field hockey. The subjects with the extended PE curriculum practiced sports only at school; participation in local or other sports clubs was a criterion for exclusion from the study.

The tests included a passive knee extension (PKE) and passive foot dorsiflexion with the knee flexed and with the knee extended. The significance of the PKE test is that the lower the angle the more flexible the hamstrings. This is because the PKE measurement is the distance to the right angle, that is a full knee extension with the hip flexed. The raters used a standard goniometer. Foot dorsiflexion and foot flexion tests with the knee bent are a good method for testing the range of motion. During the tests the ankle must be in neutral position and care must be taken to make sure that the flexion occurs at the ankle joint, not in the metatarsal or tarsal joints. The result of measurements is given in degrees. The results of the popliteal angle test are also given in degrees – the lower the result the higher the range of passive extension of the shank. The details of the studies can be found in the literature [16, 17].

The participants were examined twice by each of the 3 raters: a specialist, a resident doctor specializing in orthopaedics and a physical therapy student. The results showed good reliability and repeatability, so we decided to do the statistical analysis on the results of the first test for each of the three raters.. This is a continuation of a study on a patient cohort which we previously analyzed for other parameters. Both the grouping in the cohort and the research methods are different than in the previous studies [15, 16]. The study was approved by the Bioethics Committee at the Poznań University of Medical Sciences (approval No. 212/17). The parents gave written Informed consent and the participants – oral Informed consent to participate in the study.

A detailed description of the warm-up before PE classes is important for the topic of the paper. At the beginning of the class there was a few-minute warm-up, which included running and squats. The students also made movement of upper and lower limbs lasting less than 60 s. This was very short and did not resemble either static or dynamic stretching. On each occasion there seemed to be no plan or coordination of the warm-up. It did not resemble any preventive training programs (PTP) for any sport. There was no additional mandatory active or passive stretching at the end of the class.

The testing took place on the days when the students did not have PE classes, because such factors as the pulse, outside temperature, type of exercise, or reaching a specific temperature of the muscles could affect the measurements.

We evaluated the reliability and repeatability of the tests carried out with a 2-h interval. It was impossible to do the same type of “warm-up” so that the test carried out after 2 h would take place in exactly the same conditions. In addition, each student was each time tested by three raters. Each test took at least fifteen minutes, so the conditions for the test done by the first and the last rater would be different because the effects of warm-up would wear off. We were interested in checking natural mobility in the joints, in specific groups, reflecting the fixed range of motion.

All girls have already had their first menses. They were asked about it by a school nurse in a private setting. The stage of sexual maturity, in particular on the Tanner scale, was not assessed either for girls or for boys.

Also, there was no clinical examination of the Q-angle due to accuracy issues. In addition, as the examination starts at the anterior superior iliac spine, it would require the consent of both the parents and the subjects. We decided not to do the examination to ensure that the privacy, especially of the girls, was respected.

At the time of the study, 5117 students in total were enrolled in junior high schools in Poznań. The representativeness of the sample size was calculated using Cochran’s formula [18]. With alpha = 0.05 and the margin error of 8.1% the calculated sample size was 142.

## Results

The gender differences in the range of motion of foot dorsiflexion and the value of the popliteal angle are given in Table 1. The differences in the range of motion of foot dorsiflexion and the value of the popliteal angle between the extended PE group and the standard PE group are presented in Table 2.

**Table 1:** Gender differences in the range of motion of foot dorsiflexion and the value of the popliteal angle

Variable	Rater	Girls (*n* = 57)				Boys (*n* = 85)				p*
		$\overline{x }$±sd	median	min	max	$\overline{x }$±sd	median	min	max	
Right ankle dorsiflexion	I	7.9 ± 4.7	8	0	20	7.8 ± 5.4	8	0	20	0.850272
	II	9.7 ± 4.9	10	0	24	9.7 ± 4.6	9	0	20	0.936559
	III	8.8 ± 4.4	10	0	18	8.4 ± 4.4	10	0	20	0.378892
Left ankle dorsiflexion	I	7.6 ± 5.3	7	0	24	6.9 ± 5.5	5	0	25	0.441458
	II	10.2 ± 5.4	10	8	32	9.8 ± 4.5	10	0	22	0.853719
	III	9 ± 4.1	10	0	20	8.4 ± 3.9	10	0	20	0.649947
Right ankle dorsiflexion with knee flexion	I	18.5 ± 5.6	18	10	30	17.3 ± 6.3	18	4	35	0.267130
	II	18.5 ± 6.3	18	8	32	17.8 ± 5.6	18	4	30	0.911327
	III	25.9 ± 10.2	25	6	56	32 ± 10.2	32	12	58	0.645876
Left ankle dorsiflexion with knee flexion	I	15.5 ± 6.9	15	0	35	15.9 ± 6.5	15	0	40	0.571553
	II	17.4 ± 6.2	18	6	30	16.2 ± 6.5	14	4	32	0.262863
	III	26.9 ± 9.8	26	2	52	32.8 ± 8.7	32	12	54	0.237960
Right popliteal	I	28,4 ± 16.3	25	0	60	37.7 ± 13.8	40	8	70	**0.000531**
	II	25.5 ± 11.7	25	4	47	31.7 ± 11.5	30	14	60	**0.008744**
	III	17.5 ± 5.1	18	4	26	18.3 ± 5.4	18	8	32	**0.000677**
Left popliteal	I	32.6 ± 13.5	30	0	65	41.4 ± 12.7	42	10	70	**0.000206**
	II	26,9 ± 12.5	28	5	55	32.8 ± 10.1	32	10	60	**0.002468**
	III	17.4 ± 5.8	18	4	32	17 ± 5.1	18	6	28	**0.000285**

^*^The Student’s t-test or the Mann–Whitney U test

**Table 2:** The differences in the range of motion of foot dorsiflexion and the value of the popliteal angle between extended PE and standard PE curriculum groups

Variable	Rater	Exercisers (*n* = 82)				Non-exercisers (*n* = 60)				p*
		$\overline{x }$±sd	median	min	max	$\overline{x }$±sd	median	min	max	
Right ankle dorsiflexion	I	8.3 ± 4.9	10	0	20	7.1 ± 5.3	5.5	0	20	0.034224
	II	9.8 ± 4.7	9	0	24	9.6 ± 4.6	10	0	20	0.895814
	III	8.9 ± 4.1	10	0	18	8 ± 4.8	8	0	20	0.119661
Left ankle dorsiflexion	I	7.6 ± 5.3	8.5	0	24	6.6 ± 5.5	5	0	25	0.122463
	II	10.4 ± 4.8	10	2	22	9.3 ± 4.8	10	0	26	0.134940
	III	9.4 ± 3.7	10	0	20	7.6 ± 4.1	8	0	20	**0.009392**
Right ankle dorsiflexion with knee flexion	I	18.5 ± 6.6	18	5	35	16.7 ± 5	18	4	30	0.170130
	II	18.4 ± 6	18	6	32	17.7 ± 5.5	18	4	32	0.423305
	III	18.5 ± 5.2	18	8	32	17.2 ± 5.2	18	4	26	0.129805
Left ankle dorsiflexion with knee flexion	I	15.6 ± 6.1	15	0	35	15.9 ± 7.3	15	0	40	0.868552
	II	17.6 ± 6.4	18	6	32	15.4 ± 6.2	12	4	30	**0.034307**
	III	18 ± 5.2	18	4	32	16 ± 5.4	16	4	28	**0.031309**
Right popliteal	I	33.2 ± 15.8	35	0	60	34.9 ± 14.8	35	8	70	0.609174
	II	28.2 ± 11.8	29.5	4	60	30.6 ± 11.9	30	5	58	0.241689
	III	29.2 ± 9.8	28	10	52	30 ± 11.4	30	6	58	0.643316
Left popliteal	I	37.4 ± 13.8	40	0	70	38.7 ± 13.4	40	10	65	0.574503
	II	28.9 ± 11	29	5	55	32.6 ± 11.7	33	8	60	**0.059335**
	III	30.5 ± 9.3	30	4	52	30.7 ± 9.8	30	2	54	0.882693

^*^ The Student’s t-test or the Mann–Whitney U test

The significant differences are in bold.

### Statistical analysis

The statistical analysis was done in Statistica 12 (StatSoft Inc., Tulsa, OK, USA) and PQStat (PQStat Software, Poznań, Poland) and *p* < 0.05 was considered as statistically significant.

The non-parametric test (Mann–Whitney) was used when the variables were not normally distributed. The Student’s t-test for unpaired samples was used for normally distributed variables.

A comparison between males and females showed that the popliteal angle was significantly lower in girls**.** The same result was obtained by all the three raters, both for the right and the left leg (Table 1).

The next stage of the analysis was checking for any differences between the parameters in the groups with the extended curriculum in PE (extended PE group) and with a standard number of PE hours (standard group) (Table 2).

The statistical analysis (Mann–Whitney test and Student’s t-test, depending on data distribution) showed significant differences in the angles of right foot dorsiflexion for the first rater, and in left foot dorsiflexion for the third rater. In both cases the average values were higher in students from the extended PE groups. The second statistically significant difference found in the study was the difference between the measurements for left foot dorsiflexion with the knee bent with higher values observed in students with the extended PE curriculum (for the second and third rater). As the results were divergent we considered them to be spurious, which was later confirmed by the two-factor regression analysis.

The two-factor regression analysis for the simultaneous effect of gender and the number of PE classes (girls (57): 28 from the standard group, 31 from the extended PE group; boys (85): 34 from the standard group, 51 from the extended PE group) on the ranges of motion confirmed that the differences were observed only for the popliteal angle, which was significantly lower in girls, regardless of the level of physical activity.

The effect of female gender on the popliteal angle was shown using regression equations for two-factor analysis (gender, sport). In our studies the models showed that female gender is a statistically significant predictor of the value of the popliteal angle. For the effect of gender on the right angle the p value was 0.000287 while for the left angle – p = 0.000116. For the second rater the p values were as follows: right popliteal angle – p = 0.002286, left popliteal angle – p = 0.001511. For the third rater the models showed the effect of gender on the right angle with p = 0.000677 while for the left popliteal angle gender influence was p = 0.000285.

## Discussion

The most important findings in the literature on the impact of gender on the range of motion in the joints of the lower limbs, muscle length or flexibility are summarized below.

Several authors have found a larger flexibility of the hamstring muscles in women in different age groups [3, 19, 20]. This is consistent with our findings. However, some authors found no gender differences in this area [4].

A number of researchers highlight the differences in muscle properties between the two genders [21–24].

We have found only one paper showing that passive foot dorsiflexion is higher in women [25], which was not confirmed in our study. Other researchers [26] claim that gender does not affect foot dorsiflexion, which was also the case in our study.

There are isolated reports of a greater likelihood of increased foot dorsiflexion in women following exercise [27].

A number of researchers have highlighted the effectiveness of activity to increase the range of motion in the joints, but they either found no differences between the genders or have not examined such differences [28–32].

However, some reports suggest that activity may even decrease dorsiflexion [33].

The effect of physical activity has been discussed in a number of publications. Brugheli et al. highlight that it is possible to extend both knee flexors and extensors, as well as the triceps surae muscle [34]. The study focused on active sportspeople who exercise every day.

On the other hand, Lenskjold et al. highlight that physical activity does not influence the length of the Achilles tendon in adult runners [35]. Lichtwark et al. point out that while in in vitro tests the tendon significantly increases in length in response to stretching, the same effect has not been demonstrated in in vivo studies [36]. Physical activity may result in the thickening of the Achilles tendon while the predisposition to tendon injury may be genetically determined, irrespective of the tendon thickness [37]. It is also emphasized in the relevant literature that longer tendons, in particular the Achilles tendon, and lower muscle looseness, have beneficial effects on performance in sports [38]. According to Hirata et al., there is no evidence that stretching exercises of the triceps surae muscle can increase its length [39].

Our findings clearly show that girls between 13 and 15 years old seem to have a significantly larger hamstring flexibility, which is confirmed by the tests of the popliteal angle. Interestingly, in our study this difference seems to be unrelated to any workout or a special training programme (and in particular any PTP-based programme).

Our results also show that gender does not affect foot dorsiflexion with either extended or flexed knee.

A greater hamstring flexibility may be caused by generally higher muscle flexibility in girls in this age group (as this is the height of the PHV period). However, we did not observe the same increased flexibility in foot dorsiflexion.

The absence of statistical significance between the number of PE classes and the range of motion does not prove that exercise has no effect. However, sometimes rehabilitation is recommended to growing adolescents to manage a relative shortening of the muscles. Most often teenagers are unwilling to participate, and fail to exercise at home, the results are rather poor. In the case of the extended PE curriculum groups the decision to join was always taken by the students themselves. Their attendance in PE classes was very good.

Study limitations must be considered. The study covered only healthy individuals. In addition, the research focused on a specific age group in adolescence. However, contrary to a number of studies, our sample size was very large and representative. The measurements were not preceded by any training planned/developed to increase thigh muscle flexibility, which means that our findings point to a certain natural predisposition to hamstring lengthening and flexibility in girls in this age group.

Our results do not in any way mean that teenage girls do not need to exercise. Rather, they point to the need to focus on building balance between knee flexors and extensors in order to prevent joint injuries. This applies in particular to PTP programmes. In addition, our findings show clear sexual dimorphism in the adolescents participating in the study. Further research is needed to determine whether the larger flexibility of the hamstrings in girls is typical of a specific age group or whether it is a result of possible impact of knee alignment (as measured by the Q-angle).

## Conclusion

1. The popliteal angle, indicative of a relative shortening of the hamstrings, is larger in boys than in girls.
2. No differences were found in dorsiflexion between girls and boys who have not been trained using a training model.

## Data Availability

The datasets used and/or analysed during the current study available from the corresponding author on reasonable request.
